# Attrition in the European Child Cohort IDEFICS/I.Family: Exploring Associations Between Attrition and Body Mass Index

**DOI:** 10.3389/fped.2018.00212

**Published:** 2018-08-15

**Authors:** Malte Langeheine, Hermann Pohlabeln, Fabio Lauria, Toomas Veidebaum, Michael Tornaritis, Denes Molnar, Gabriele Eiben, Stefaan de Henauw, Luis A. Moreno, Garrath Williams, Wolfgang Ahrens, Stefan Rach

**Affiliations:** ^1^Leibniz Institute for Prevention Research and Epidemiology–BIPS, Bremen, Germany; ^2^Institute of Food Sciences, National Research Council, Avellino, Italy; ^3^National Institute for Health Development, Tallinn, Estonia; ^4^Research and Education Institute of Child Health, Strovolos, Cyprus; ^5^Department of Pediatrics, Medical School, University of Pécs, Pécs, Hungary; ^6^Section for Epidemiology and Social Medicine, Sahlgrenska Academy, University of Gothenburg, Gothenburg, Sweden; ^7^Department of Biomedicine and Public Health, School of Health and Education, University of Skövde, Skövde, Sweden; ^8^Department of Public Health, Ghent University, Ghent, Belgium; ^9^Growth, Exercise, Nutrition and Development, Research Group, Faculty of Health Sciences, University of Zaragoza, Instituto Agroalimentario de Aragón, Instituto de Investigación Sanitaria de Aragón and Centro de Investigación Biomédica en Red de Fisiopatología de la Obesidad y Nutrición, Zaragoza, Spain; ^10^Department of Politics, Philosophy and Religion, Lancaster University, Lancaster, United Kingdom; ^11^Faculty of Mathematics and Computer Science, Institute of Statistics, University Bremen, Bremen, Germany

**Keywords:** cohort attrition, child health, BMI, selection effects, cross country differences

## Abstract

Attrition may lead to bias in epidemiological cohorts, since participants who are healthier and have a higher social position are less likely to drop out. We investigated possible selection effects regarding key exposures and outcomes in the IDEFICS/I.Family study, a large European cohort on the etiology of overweight, obesity and related disorders during childhood and adulthood. We applied multilevel logistic regression to investigate associations of attrition with sociodemographic variables, weight status, and study compliance and assessed attrition across time regarding children's weight status and variations of attrition across participating countries. We investigated selection effects with regard to social position, adherence to key messages concerning a healthy lifestyle, and children's weight status. Attrition was associated with a higher weight status of children, lower children's study compliance, older age, lower parental education, and parent's migration background, consistent across time and participating countries. Although overweight (odds ratio 1.17, 99% confidence interval 1.05–1.29) or obese children (odds ratio 1.18, 99% confidence interval 1.03–1.36) were more prone to drop-out, attrition only seemed to slightly distort the distribution of children's BMI at the upper tail. Restricting the sample to subgroups with different attrition characteristics only marginally affected exposure-outcome associations. Our results suggest that IDEFICS/I.Family provides valid estimates of relations between socio-economic position, health-related behaviors, and weight status.

## Introduction

Epidemiological cohort studies are not only prone to non-response at baseline, but also to drop-out of participants during follow-up (1), called cohort attrition. Since non-response and drop-out are more likely among less healthy and disadvantaged study participants (2–4), it is especially important for cohort studies to assess selection effects. IDEFICS (Identification and prevention of dietary and lifestyle-induced health effects in children and infants) (5) and I.Family (IDEFICS/I.Family cohort) (6) is a large European prospective cohort including children from eight countries (Belgium, Cyprus, Estonia, Germany, Hungary, Italy, Spain, and Sweden) that has been investigating dietary, behavioral and socioeconomic factors in relation to non-communicable chronic diseases and disorders with a focus on overweight and obesity (5, 6). In IDEFICS/I.Family, a total of 16,228 children and their parents took part in up to three physical examinations between 2007 and 2014 and completed questionnaires on medical history, dietary behavior and other aspects of children's life. The present analysis complements the IDEFICS/I.Family cohort profile (5, 6). We extend the attrition analysis that included only the first follow-up examination (7) and we build on the observed selection effects at baseline (8) and the association between recruitment effort and drop-out (9). Here we investigate the association of cohort attrition with sociodemographic characteristics, weight status, and study compliance in IDEFICS/I.Family (“study compliance” marks how far child and parents undertook all the requested measures and questionnaires). We also consider variations of attrition across the first and second follow-up and between the participating countries, focusing on selection effects by children's weight status.

## Methods

### Analysis group

In IDEFICS/I.Family, data were collected in each country in two or more selected communities. The sociodemographic profile and infrastructure of the communities were similar and typical for their region. All children aged 2–9.9 years attending kindergarten or primary school within each community were eligible. Parents of potential study subjects were either approached directly by mail or by letters delivered through teachers and caretakers in kindergartens and schools. They were asked for consent to examine their children as well as to answer a number of questionnaires. Children and parents were informed about all aspects of the study. Parents gave their written informed consent prior to inclusion into the study; children 12 years or older signed a simplified consent form. Immediately before each examination, a study nurse informed each child orally about the module using a simplified preformulated text. Children were informed that they do not have to participate if they don't want to and examinations were only performed if children assented and parents consented. Consent could be given to single components of the study while refusing others.

All procedures performed in IDEFICS/I.Family were in accordance with the ethical standards of the institutional committee and the 1964 Declaration of Helsinki and its later amendments. Approval was obtained by each of the centers engaged in the fieldwork by its appropriate ethics committees. To ensure that data collection and study parameters were similar between countries, a common manual of operations containing standard operating procedures for all examinations was developed, and site visits were conducted in all study centers by a central quality control to ensure compliance.

**Figure 1:**
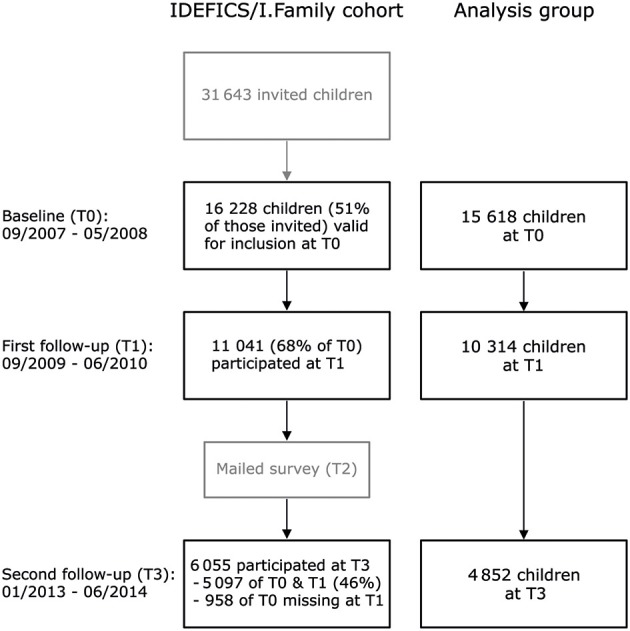
Flow chart of the participation in the IDEFICS baseline examination (T0), the first IDEFICS follow-up examination (T1), and the second follow-up examination in the I.Family study (T3). The refreshment samples at the first and the second follow-up (6) were excluded from this analysis.

In total, 16,228 children participated in the IDEFICS baseline examination (T0), carried out between September 2007 and May 2008 (Figure 1). All children who took part in the baseline examination were invited to the first follow-up (T1) between September 2009 and May 2010 where 11,041 children participated. Baseline and first follow-up included identical examination modules.

A second follow-up examination (I.Family, T3) was conducted between 2013 and 2014, again with similar examination modules (6). Children who participated at baseline, their siblings, and their parents were invited to take part in I.Family, and a total of 6,055 IDEFICS children were examined. Of the 11,041 children examined at the first follow-up, 5,097 children took part in I.Family. In addition, 958 children took part in I.Family who participated at baseline, but not in the first follow-up. Due to model constraints, these children were considered first follow-up drop-outs, that is, only baseline data were included in the analysis. A complete-cases analysis reduced the sample size to 15,618 children at baseline, 10,314 children at the first follow-up, and 4,852 children at the second follow-up. This resulted in a total of 25,932 person-wave observations at baseline and the first follow-up being included in the analysis. Baseline characteristics of the IDEFICS/I.Family baseline sample and the subsamples that participated in the two follow-ups are summarized in Table 1.

**Table 1:** Baseline characteristics of the IDEFICS/I.Family baseline sample and the subsamples that participated in the two follow-ups.

			**Participated at T1**				**Participated at T3**			
	**Baseline participants**		**Yes**		**No**		**Yes**		**No**	
	**Mean**	**SD**	**Mean**	**SD**	**Mean**	**SD**	**Mean**	**SD**	**Mean**	**SD**
Age child (years)	6.0	1.8	6.0	1.8	6.0	1.8	5.9	1.8	6.1	1.8
Compliance score of child	6.3	1.1	6.4	1.1	6.2	1.3	6.4	1.0	6.4	1.1
Compliance score parent(s)	3.5	0.8	3.5	0.8	3.5	0.8	3.5	0.7	3.4	0.8
Mother's age (years)	35.2	5.3	35.5	5.3	34.5	5.4	35.7	5.2	35.4	5.3
	***n***	**%**	***n***	**%**	***n***	**%**	***n***	**%**	***n***	**%**
Sex of child										
Male	7,928	50.8	5,418	50.6	2,510	51.1	2,511	50.8	2,907	50.4
Female	7,690	49.2	5,291	49.4	2,399	48.9	2,433	49.2	2,858	49.6
Weight status child										
Normal weight	12,543	80.3	8,724	81.5	3,819	77.8	4,002	80.9	4,722	81.9
Overweight	1,963	12.6	1,275	11.9	688	14.0	611	12.4	664	11.5
Obese	1,112	7.1	710	6.6	402	8.2	331	6.7	379	6.6
Weight status parents										
No parent overweight	3,943	25.2	2,836	26.5	1,107	22.6	1,355	27.4	1,481	25.7
At least one parent overweight	10,059	64.4	6,909	64.5	3,150	64.2	3,162	64.0	3,747	65.0
Missing	1,616	10.3	964	9.0	652	13.3	427	8.6	537	9.3
Migration background										
No	12,906	82.6	8,972	83.8	3,934	80.1	4,149	83.9	4,823	83.7
Partly	1,384	8.9	939	8.8	445	9.1	475	9.6	464	8.0
Full	1,079	6.9	656	6.1	423	8.6	265	5.4	391	6.8
Missing	249	1.6	142	1.3	107	2.2	55	1.1	87	1.5
Educational level										
Low education	7,335	47.0	5,387	50.3	1,948	39.7	2,582	52.2	2,805	48.7
Medium education	7,010	44.9	4,554	42.5	2,456	50.0	2,062	41.7	2,492	43.2
High education	1,073	6.9	651	6.1	422	8.6	251	5.1	400	6.9
Missing	200	1.3	117	1.1	83	1.7	49	1.0	68	1.2
Number of adults in household										
One	1,255	8.0	763	7.1	492	10.0	332	6.7	431	7.5
Two	11,032	70.6	7,847	73.3	3,185	64.9	3,633	73.5	4,214	73.1
Three	1,081	6.9	740	6.9	341	6.9	372	7.5	368	6.4
Four or more	424	2.7	270	2.5	154	3.1	134	2.7	136	2.4
Missing	1,826	11.7	1,089	10.2	737	15.0	473	9.6	616	10.7
Siblings aged < 18 years										
Yes	3,667	23.5	2,364	22.1	1,303	26.5	1,063	21.5	1,301	22.6
No	10,364	66.4	7,412	69.2	2,952	60.1	3,482	70.4	3,930	68.2
Missing	1,587	10.2	933	8.7	654	13.3	399	8.1	534	9.3
Region										
Intervention	8,075	51.7	5,525	51.6	2,550	51.9	2,607	52.7	2,918	50.6
Control	7,543	48.3	5,184	48.4	2,359	48.1	2,337	47.3	2,847	49.4
Country										
Spain	1,480	9.5	1,203	11.2	277	5.6	429	8.7	774	13.4
Hungary	2,496	16.0	1,218	11.4	1,278	26.0	468	9.5	750	13.0
Germany	2,008	12.9	1,165	10.9	843	17.2	637	12.9	528	9.2
Cyprus	2,111	13.5	1,589	14.8	522	10.6	862	17.4	727	12.6
Estonia	1,650	10.6	1,284	12.0	366	7.5	721	14.6	563	9.8
Belgium	1,884	12.1	1,236	11.5	648	13.2	242	4.9	994	17.2
Italy	2,241	14.3	1,543	14.4	698	14.2	873	17.7	670	11.6
Sweden	1,748	11.2	1,471	13.7	277	5.6	712	14.4	759	13.2
*N*	15,618	100	10,709	100	4,909	100	4,944	100	5,765	100

*Note that percentages may not sum to 100% due to rounding. SD, standard deviation*.

### Outcome

The outcome *cohort attrition* was defined with respect to participation in the first (T1) and the second (T3) follow-up examination (0: participation vs. 1: dropout).

### Exposures

The social position of families was classified according to the International Standard Classification of Education (ISCED) (10) using the highest educational attainment of mother or father (low: ISCED levels 0–2; medium: ISCED levels 3–4; high: ISCED levels 5 and higher). The household composition was described as the presence of non-adult siblings besides the participating child (yes vs. no) and the number of adults (age 18 or older) living in the household. The place of birth of parents served to define the migration background (full migrant: both parents foreign-born; partly migrant: one parent foreign-born; not migrant: otherwise). Children's age and mother's age on the day of the examination was recorded in years. For drop-outs at the first or second follow-up, children's, and mother's age was estimated by adding the mean duration between two examinations to the age at the previous examination. Because of collinearity and a higher percentage of missing values, the father's age was not considered in the analysis. The weight status was determined using the body mass index (BMI). Children's weight status (thin and normal weight, overweight, obese) was categorized according to Cole and Lobstein (11). Parent's weight status (self-reported) was categorized as “no parent overweight,” “at least one parent overweight,” and “missing.” Overweight was defined as having a BMI ≥25. A score of study compliance was constructed separately for children and parents based on the number of key examination modules they participated in at baseline and at first follow-up (Table 2). This was done by counting the number of completed modules (0: module not completed; 1: module completed). For children, key modules were blood pressure, bioelectrical impedance analysis (fasting state), waist-to-hip ratio, skinfold thickness (subscapularis and triceps), blood sample (fasting state), morning urine, and saliva. Parent's (respectively mother or father) provided key modules included the general questionnaire, food frequency questionnaire, medical history, and the 24-h dietary recall. At the first follow-up, the collection of saliva was restricted to children without a saliva sample at baseline. Therefore saliva was defined as being available at first follow-up if a sample was available at baseline or first follow-up.

**Table 2:** Number and percentage of children and parents participating in examination modules at baseline and at first follow-up.

	**Examination module**	**Participation**	
		**Baseline *n* (%)**	**First follow-up *n* (%)**
Children	Blood pressure	14,752 (90.9)	10,563 (95.7)
	Bioelectrical impedance analysis (fasting state)	15,720 (96.9)	10,795 (97.8)
	Waist-to-hip ratio	15,551 (95.8)	10,731 (97.2)
	Skinfold thickness (subscapularis and triceps)	15,160 (93.4)	10,567 (95.7)
	Venous or capillary blood (fasting state)	12,855 (79.2)	8,528 (77.2)
	Morning urine	13,945 (85.9)	8,845 (80.1)
	Saliva^a^	14,273 (88.0)	188 (1.7)
Parents	General questionnaire (children parents)	16,117 (99.3)	10,539 (95.5)
	Food frequency questionnaire	15,199 (93.7)	9,963 (90.2)
	Medical history	12,418 (76.5)	8,978 (81.3)
	24-h dietary recall	11,671 (71.9)	5,520 (50.0)

a *The collection of saliva at the first follow-up was restricted to children without a saliva sample at baseline. Therefore saliva was defined as being available at first follow-up if a sample was available at baseline or first follow-up*.

### Statistical analysis

The association between attrition and sociodemographic variables, weight status, and study compliance was assessed by estimating odds ratios (ORs) and 99% confidence intervals (CIs) using a multivariable multilevel logistic regression with respondents as the second-level variable and country as the third-level to account for clustering (12). To avoid that meaningless associations become statistically significant just because of the large sample size and to account for multiple testing of associations a more stringent criterion for statistical significance (α = 0.01) was chosen. Data were transformed such that each unit of analysis represented a person-wave observation (13, 14). Variables included in the model were either time constant (e.g., sex of the child), or time-variant predictors (e.g., weight status of the child). Time-variant predictors were modeled as lagged covariates, that is, information at baseline was regressed on attrition at first follow-up and information at the first follow-up was regressed on attrition at the second follow-up. Sensitivity analyses were carried out to check for non-independence of siblings in the sample. Random sampling (*n* = 100) was used to select one child of each family and calculate a random intercept logistic regression model for each sample to obtain a mean odds ratio and a corresponding confidence interval for each predictor. The odds ratios of a logistic regression model with all children and the mean odds ratios for the 100 samples did not differ substantially. To assess the variation of attrition across time in separate models all possible interaction terms between potential predictors of attrition and time point of follow-up examination were calculated [time × (sex of child, age child, weight status child, compliance score of child, compliance score parent(s), mother's age, weight status parents, migration background, educational level, number of adults in household, siblings aged <18 years, and region)]. The heterogeneity between the countries was investigated by means of meta-analyses: Country-stratified logistic regression models with attrition as the dependent variable and the same predictors as in the random intercept logistic regression model were fitted and a random-effects meta-analysis (RE model) (15) was calculated for each predictor of the country-stratified logistic regression models. To evaluate the heterogeneity of attrition between the countries, the percentage of variation that is due to heterogeneity, *I*^2^ (16), and forest plots were used. Selection effects on children's BMI across time were assessed with quantile-quantile plots (Q-Q plots) and Kolmogorov-Smirnov tests (KS test) (17). We explored the impact of selection effects on the cross-sectional association of social position and weight status. Children's weight status was converted into a binary variable (0: normal weight including thin vs. 1: obese including overweight) further referred to as overweight/obesity. Social position included educational level (as described above) and income level (low, low/medium, medium, medium/high, vs. high income). We estimated baseline associations and then estimated identical associations with subsamples restricted to first follow-up participants (T1) and second follow-up participants (T3) as well as associations at the first follow-up (T1) and the restricted sample of second follow-up participants (T3). In addition, we explored selection effects on the association between adherence to key messages of a healthy lifestyle promoted by IDEFICS/I.Family and overweight/obesity published by Kovacs et al. (18). In this analysis we included total screen time, moderate to vigorous physical activity (MVPA), and sleep duration as measures of adherence [see (18) for detailed information on instruments and operationalization]. In accordance with Kovacs et al. (18), we calculated a binary indicator for adherence on respective cut points for screen time, MVPA, and sleep. We estimated the baseline association of adherence and overweight/obesity and then estimated the identical association with subsamples restricted to first follow-up participants (T1) and second follow-up participants (T3). For the exposure-outcome association of adherence to key messages of a healthy lifestyle and overweight/obesity, as well as social position and overweight/obesity we estimated odds ratios and confidence intervals with multivariable multilevel logistic regression models. For the sake of comparability we used 95% confidence intervals in the analysis reproducing the association of adherence to key messages and overweight/obesity published by Kovacs et al. (18) (described above). All other analyses, as pointed out above, utilized 99% confidence intervals.

To quantify a potential bias we calculated the percent change in point estimates (CPE = OR _subsample_/OR _full_ _sample_ × 100 – 100). We considered a CPE of above 10% as indicator of a bias. For Table 6 we stratified overweight/obesity by the combination of adherence to key messages regarding media consumption, physical activity and sleep. Children who *did not adhere* to the recommendations of screen time and physical activity and sleep duration were assigned to the group – – – (1,666 children in T0 full sample). In contrast, children who *did adhere to all* recommendations of screen time and physical activity and sleep duration were assigned to the group + + + (263 children in T0 full sample). Children who adhered only to some of the recommendations were assigned accordingly. A full description of the analysis is given in Kovac et al. (18). Analyses were performed using R version 3.3.3 (http://www.r-project.org/).

## Results

The multilevel logistic regression model with cohort attrition as dependent variable (Table 3) revealed that children's age in years was positively associated with attrition (OR 1.05, 99% CI 1.02–1.07). Compared to normal weight children, overweight (OR 1.17, 99% CI 1.05–1.29) or obese (OR 1.18, 99% CI 1.03–1.36) children had a higher chance of attrition. Higher study compliance of children was associated with lower attrition (OR 0.84, 99% CI 0.81–0.87), as was higher mother's age (OR 0.98, 99% CI 0.97–0.99). Children with a partly (OR 1.13, 99% CI 1.00–1.28) or full migrant background (OR 1.41, 99% CI 1.21–1.63) had a higher chance of attrition, as had children of parents with a low (OR 1.49, 99% CI 1.27–1.74) or medium (OR 1.19, 99% CI 1.10–1.29) educational level.

**Table 3:** Odds ratios with 99% confidence intervals for cohort attrition.

	**Cohort attrition**				
	**No**		**Yes**		**OR^a^ (99% CI)**
	***n***	**%**	***n***	**%**	
Time					
First follow-up (T1)	10,709	68.6	4,909	31.4	ref.
Second follow-up (T3)	4,852	47.0	5,462	53.0	2.62 (2.32–2.96)
Sex of child^b^					
Male	7,883	60.1	5,244	39.9	ref.
Female	7,678	60.0	5,127	40.0	0.99 (0.93–1.07)
Age child (years)^c^					1.05 (1.02–1.07)
Weight status child^c^					
Normal weight	12,418	60.9	7,978	39.1	ref.
Overweight	2,059	56.5	1,588	43.5	1.17 (1.05–1.29)
Obese	1,084	57.4	805	42.6	1.18 (1.03–1.36)
Compliance score of child^c^					0.84 (0.81–0.87)
Compliance score parent(s)^c^					0.93 (0.88–0.98)
Mother's age (years)^c^					0.98 (0.97–0.99)
Weight status parents^c^					
No parent overweight	4,010	62.7	2,388	37.3	ref.
At least one parent overweight	10,074	60.2	6,660	39.8	1.05 (0.96–1.14)
Missing	1,477	52.8	1,323	47.2	1.23 (1.08–1.40)
Migration background^b^					
No	12,961	60.6	8,427	39.4	ref.
Partly	1,395	61.6	871	38.4	1.13 (1.00–1.28)
Full	905	53.8	778	46.2	1.41 (1.21–1.63)
Missing	300	50.4	295	49.6	1.35 (0.96–1.91)
Educational level^b^					
Low education	7,965	63.2	4,639	36.8	1.49 (1.27–1.74)
Medium education	6,656	57.9	4,837	42.1	1.19 (1.10–1.29)
High education	912	53.4	796	46.6	ref.
Missing	28	22.0	99	78.0	1.12 (0.77–1.62)
Number of adults in household^c^					
One	1,122	54.2	949	45.8	ref.
Two	11,401	61.5	7,149	38.5	0.89 (0.78–1.02)
Three	1,109	59.5	755	40.5	0.98 (0.82–1.18)
Four or more	414	57.3	308	42.7	1.02 (0.80–1.30)
Missing	1,515	55.6	1,210	44.4	1.02 (0.72–1.44)
Siblings aged < 18 years^c^					
Yes	10,897	61.6	6,796	38.4	0.90 (0.83–0.99)
No	3,334	57.3	2,480	42.7	ref.
Missing	1,330	54.8	1,095	45.2	0.94 (0.67–1.34)
Region^b^					
Intervention	8,072	60.3	5,322	39.7	ref.
Control	7,489	59.7	5,049	40.3	1.05 (0.98–1.13)
Country (third-level)^b^					
Spain	1,622	61.4	1,020	38.6	
Hungary	1,622	46.5	1,868	53.5	
Germany	1,808	57.2	1,352	42.8	
Cyprus	2,455	66.2	1,252	33.8	
Estonia	2,009	68.4	927	31.6	
Belgium	1,480	48.1	1,597	51.9	
Italy	2,414	63.8	1,368	36.2	
Sweden	2,151	68.5	987	31.5	
*N*	15,561	60.0	10,371	40.0	

*OR, odds ratio; CI, confidence interval; ref., reference category*.

a *Adjusted for country*.

b *Time invariant variable using information from baseline (T0)*.

c *Time variant variable using information from baseline (T0) and first follow-up (T1)*.

**Figure 2:**
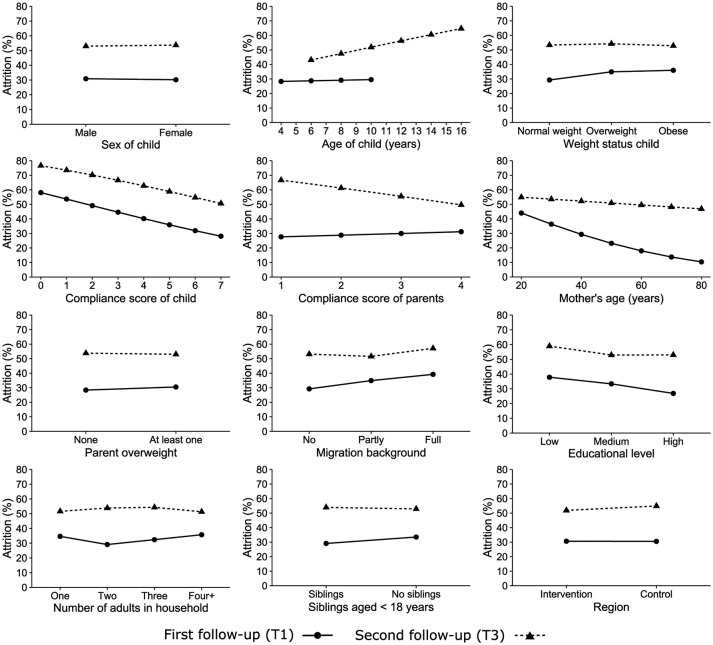
Predicted probabilities of separately modeled interactions of the predictors and time in the random intercept logistic regression. Time variant variables are age of child (years), weight status of child, compliance score of child and of parents, mother's age (years), and parent's overweight.

Variations of attrition across time are depicted in Figure 2 as probabilities predicted from separate random intercept logistic regression models containing interaction terms between potential predictors of attrition and time point of follow-up examination. Age of child was not associated with attrition at the first follow-up but was positively associated with attrition at the second follow-up. Higher parent's study compliance was associated with lower attrition at the second follow-up, but was not associated with attrition at the first follow-up. A higher age of the mother was associated with lower attrition at first follow-up but not at the second follow-up.

**Figure 3:**
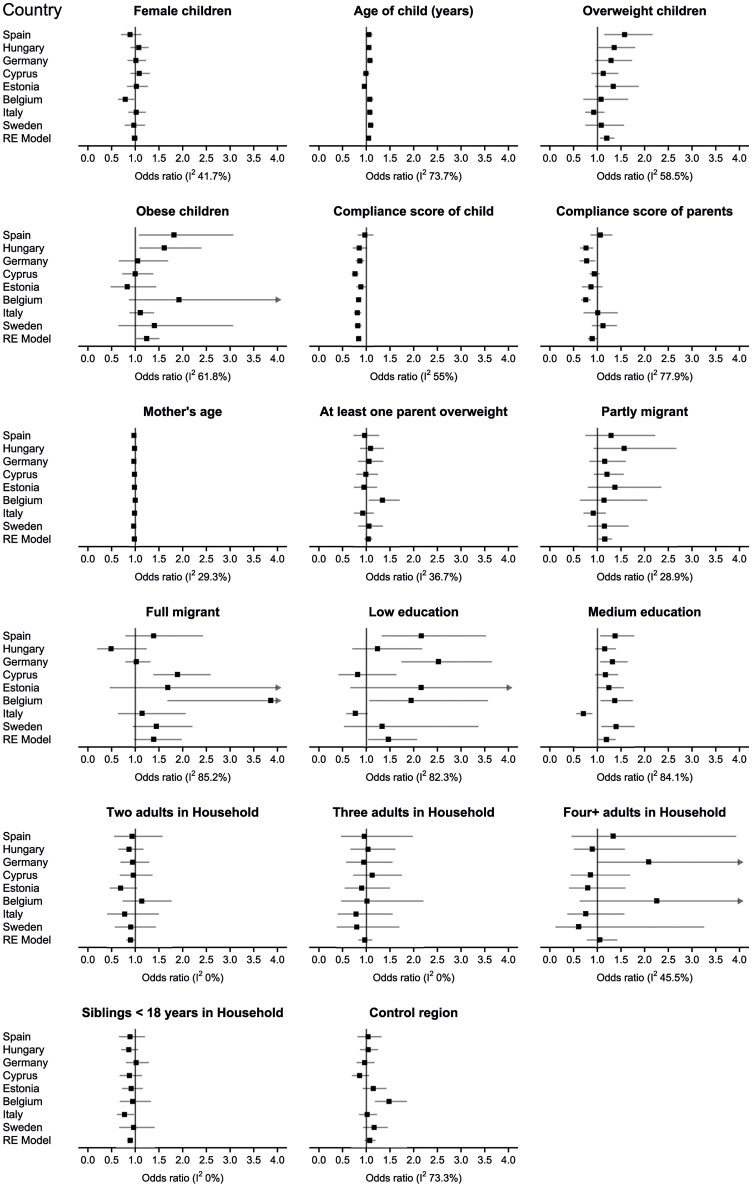
Odds ratios for attrition (with 99% confidence intervals, CI) from country-stratified logistic regression models (ordered by baseline response) with attrition as dependent variable and same predictors as in the random intercept logistic regression model (Table 3) as well as a pooled estimate of a random-effects meta-analyses (RE model), and *I*^2^ (%) as a measure of heterogeneity between the countries. *I*^2^ values above 50% indicating substantial heterogeneity were observed for 9 out of 17 variables. Arrows at the upper limit of a CI: SD, standard deviation confidence interval extends past four.

To assess how well the model represented data of individual countries, we explored with forest plots whether single countries differed notably from the overall pattern, that is, whether the sign of a countries' odds ratio for a given exposure variable differed from the pooled estimate (Figure 3). For 14 out of 17 predictors, estimates for all countries were in line with the pooled estimate. Female children in Belgium had a lower chance of attrition, whereas no association of sex was found for the pooled estimate. A medium educational level was associated with a lower chance of attrition in Italy, while the pooled estimate indicated a higher chance of attrition for a low or high educational level. Further, children from the control region in Belgium had a higher chance of attrition while no association for the region was evident in the pooled estimate. Between countries, substantial heterogeneity was observed for study compliance of children, weight status (overweight/obese; *I*^2^ from 50 to 70%), age of the child, study compliance of parents, full migrant status, low or medium education, and control region (*I*^2^ from 70 to 100%). Sensitivity analyses showed that exclusion of country-stratified odds ratios identified as exceptions attenuated *I*^2^: Excluding Belgium decreased *I*^2^ to zero for the predictor female and decreased *I*^2^ for the control region; excluding Italy decreased the *I*^2^ of low education.

**Figure 4:**
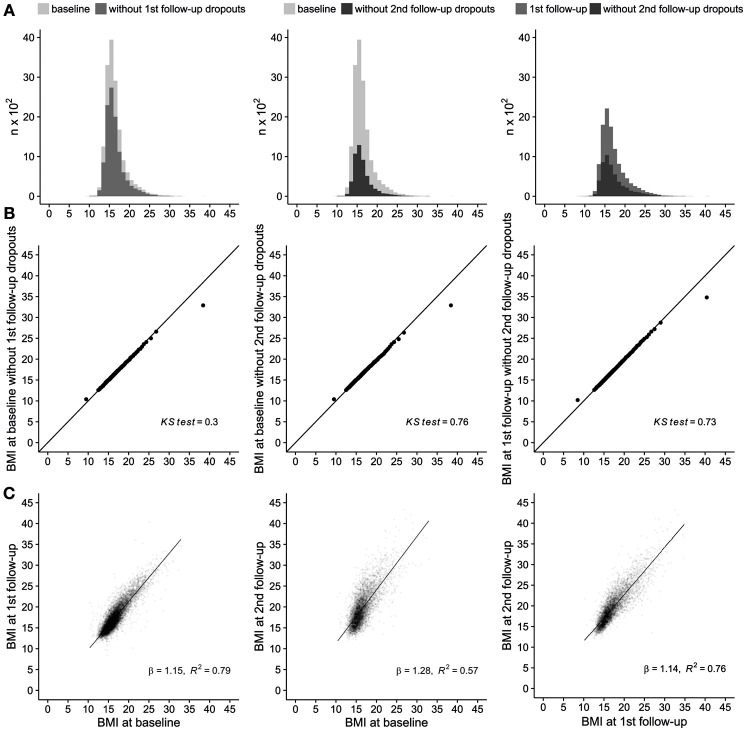
Effect of cohort attrition on the distribution of children's body mass index (BMI). **(A)** Histograms of children's BMI at baseline for different subgroups show no evidence for differences in distributions. Left: full baseline sample vs. baseline sample without attrition at first follow-up; middle: full baseline sample vs. baseline sample without attrition at second follow-up; right: first follow-up sample vs. first follow-up sample without attrition at second follow-up. **(B)** Quantile-quantile plot (Q-Q plot) and Kolmogorov-Smirnov test (KS test, 2 sided *P* value) of children's baseline BMI for different subgroups show no evidence for differences in distributions. Results of KS tests (all *p* ≥ 0.3) also indicate insufficient evidence to reject the null hypothesis that the respective distributions are the same. Left: full baseline sample vs. baseline sample without attrition at first follow-up; middle: full baseline sample vs. baseline sample without attrition at second follow-up; right: first follow-up sample vs. first follow-up sample without attrition at second follow-up. **(C)** Scatter plot and results of linear regressions (ß coefficients and *R*^2^) between children's BMI at different time points indicate that the correlation was consistent across time. Left: full baseline sample vs. baseline vs. first follow-up, middle: baseline vs. second follow-up; right: first follow-up vs. second follow-up.

Since IDEFICS/I.Family was a multi-purpose cohort focusing on overweight and obesity, we further investigated selection effects of children's BMI. BMI distributions for all children and the corresponding BMI distributions for children that did not drop out at a particular follow-up are displayed in Figure 4, column 1–3. The histograms of BMI at baseline and BMI at baseline without the children that dropped out at the first follow-up differed in the number of observations per bin but the Q-Q plot as well as the KS test (2 sided *P*-value of 0.30) (Figure 4B, column 1) indicated equal distributions. Similar results were obtained for the distribution of BMI at the first follow-up and the resulting distribution when second follow-up drop-outs were excluded (KS test: 2 sided *P*-value of 0.73) (Figure 4B, column 2) as well as for the distribution of BMI at baseline and the corresponding distribution without second follow-up dropouts (KS test: 2 sided *P*-value of 0.76) (Figure 4B, column 3).

Density scatter plots with children's BMI at baseline plotted against BMI at the first follow-up (respectively BMI at first follow-up vs. BMI at second follow-up; BMI at baseline vs. BMI at second follow-up) and ß coefficients of linear regression models were used to evaluate selection effects of BMI across time (Figure 4C). The correlation between children's BMI at different time points was consistent across time, both in the shape of the scatter plot and the ß coefficients (baseline vs. first follow-up: ß = 1.15, *R*^2^ = 0.79; first follow-up vs. second follow-up: ß = 1.14, *R*^2^ = 0.76; baseline vs. second follow-up: ß = 1.28, *R*^2^ = 0.57).

We explored the impact of selection effects due to the association between childhood overweight and social position [e.g., (19); for a review, (20)] at baseline and both follow-ups. To this end, we estimated associations between BMI and social variables in the complete baseline sample and compared them to associations between the same variables in two subsamples restricted to participants of the first follow-up and participants of the second follow-up, respectively (Table 4). We repeated this procedure with data from the first follow-up for all participants of the first follow-up and a subsample restricted to all participants of the second follow-up (Table 4).

**Table 4:** Association between overweight/obesity and social position (odds ratios with 99% confidence intervals).

	**Baseline association**					**First follow-up association**		
	**T0 full sample**	**T1 subsample**	**% change in**	**T3 subsample**	**% change in**	**T1 full sample**	**T3 subsample**	**% change in**
	**OR^a^ (99% CI)**	**OR^a^ (99% CI)**	**OR (T1 vs. T0)**	**OR^a^ (99% CI)**	**OR (T3 vs. T0)**	**OR^a^ (99% CI)**	**OR^a^ (99% CI)**	**OR (T3 vs. T1)**
Income level^c^								
Low	1.43 (1.12–1.82)	1.34 (1.00–1.81)	−6.3	1.55 (1.02–2.38)	8.4	1.47 (1.13-1.92)	1.79 (1.22-2.63)	21.8
Low/medium	1.38 (1.09–1.74)	1.36 (1.02–1.80)	−1.5	1.33 (0.87–2.03)	−3.6	1.43 (1.12–1.84)	1.77 (1.23–2.57)	23.8
Medium	1.36 (1.10–1.68)	1.30 (1.01–1.68)	−4.4	1.51 (1.04–2.19)	11.0	1.39 (1.12–1.73)	1.53 (1.11–2.11)	10.1
Medium/high	1.25 (1.00–1.57)	1.18 (0.90–1.55)	−5.6	1.29 (0.86–1.92)	3.2	1.16 (0.92–1.48)	1.40 (0.98–1.98)	20.7
High	ref.	ref.	ref.	ref.	ref.	ref.	ref.	ref.
Educational level^b^								
Low	1.55 (1.21–1.97)	1.60 (1.18–2.17)	1.6	1.78 (1.12–2.84)	9.0	1.72 (1.27–2.32)	1.69 (1.06–2.69)	−8.2
Medium	1.22 (1.06–1.40)	1.24 (1.05–1.47)	3.2	1.33 (1.04–1.70)	14.8	1.22 (1.04–1.43)	1.12 (0.88–1.41)	−1.7
High	ref.	ref.	ref.	ref.	ref.	ref.	ref.	ref.
Age child (years)^c^	1.24 (1.20–1.29)	1.25 (1.20–1.31)	0.8	1.23 (1.16–1.31)	−0.8	1.18 (1.14–1.23)	1.19 (1.12–1.26)	0.9
Sex of child^b^								
Male	ref.	ref.	ref.	ref.	ref.	ref.	ref.	ref.
Female	1.21 (1.07–1.36)	1.20 (1.04–1.39)	−0.8	1.14 (0.92–1.40)	−5.8	1.11 (0.97–1.27)	1.14 (0.93–1.38)	2.7
Region^b^								
Intervention	ref.	ref.	ref.	ref.	ref.	ref.	ref.	ref.
Control	0.94 (0.84–1.06)	0.95 (0.82–1.10)	1.1	0.89 (0.72–1.09)	−5.3	0.97 (0.84–1.11)	0.89 (0.73–1.09)	−8.3
*N*	13,855	9,604		4,461		9,068	4,340	

*OR, odds ratio; CI, confidence interval; ref., reference category*.

a *Odds ratios and confidence intervals for overweight/obesity adjusted for country*.

b *Time invariant variable using information from baseline (T0)*.

c *Time variant variable using information from baseline (T0), first follow-up (T1) or second follow-up (T3)*.

At all time points, a lower income level was associated with a higher chance of overweight/obesity. Restricting the baseline association (T0) of income level to T1 participants marginally affected odds ratios indicated by a CPE of <10% but led to bigger confidence intervals [e.g., low income at baseline (T0): full sample (OR 1.43, 99% CI 1.12–1.82) vs. T1 participants (OR 1.34, 99% CI 1.00–1.81) vs. T3 participants (OR 1.55, 99% CI 1.02–2.38)]. A restriction to T3 participants resulted in a CPE for medium income level of 11%. For the restricted subsample at first follow-up (T1 full sample restricted to T3 participants), odds ratios tended to be higher as compared to other estimates of income level. Apart from medium income, CPE of income level was well above 10%.

A lower educational level was associated with a higher chance of overweight/obesity at baseline and first follow-up. Restricting the association of educational level and overweight/obesity did not affect the trend of this association, with a CPE for the baseline association restricted to T3 participants of 14.8% for medium educational level and the confidence intervals.

IDEFICS/I.Family covered multiple topics including diet, physical activity, sleep, and stress. Results from the baseline examination showed that adherence to key behaviors of a healthy lifestyle was associated with a lower chance of overweight/obesity (18). We checked whether BMI related selection effects changed the results of Kovacs et al. (18) if the sample was restricted to participants of the first follow-up and the second follow-up, respectively. At baseline, adherence to the key messages total screen time, MVPA and sleep duration (Table 5) was associated with a lower chance of overweight/obesity [OR 0.76, 95% CI 0.70–0.83; OR 0.70, 95% CI 0.58–0.84; OR 0.85, 95% CI 0.74–0.96; reported in Kovacs et al. (18), their Table 4, rightmost column]. Estimating the association of key messages and overweight/obesity restricted to a subsample of T1 participants affected odds ratios and confidence intervals marginally with a CPE of <5%. But a restriction to a subsample of T3 participants resulted in confidence intervals for MVPA and sleep duration that included the reference category. Nevertheless, only the CPE for MVPA (22.9%) exceeded 10%.

**Table 5:** Association between overweight/obesity and adherence to key messages concerning a healthy lifestyle (odds ratios with 95% confidence intervals).

	**Baseline association**				
	**T0 full sample**	**T1 subsample**	**% change in**	**T3 subsample**	**% change in**
	**OR^a^ (95% CI)**	**OR^a^ (95% CI)**	**OR (T1 vs. T0)**	**OR^a^ (95% CI)**	**OR (T3 vs. T0)**
Total screen time^b^	0.76 (0.70–0.83)	0.79 (0.71–0.88)	4.0	0.80 (0.68–0.93)	5.3
N	15,084	10,374		4,791	
MVPA >60 min per day^c^	0.70 (0.58–0.84)	0.69 (0.55–0.86)	−1.4	0.86 (0.61–1.20)	22.9
N	7,447	5,219		2,421	
Sleep duration^d^	0.85 (0.74–0.96)	0.82 (0.70–00.96)	−3.5	0.90 (0.72–1.14)	5.9
N	10,495	7,370		3,562	

*OR, odds ratio; CI, confidence interval*.

a *Odds ratios and confidence intervals for overweight/obesity of single models adjusted for age, sex, and country*.

b *<1 h in pre-school and <2 h in school children (reference category: ≥1 h in pre-school and ≥2 h in school children)*.

c *Reference category: MVPA ≤ 60 min per day*.

d *≥11 h in pre-school and ≥10 h in school children (reference category: <11 h in pre-school and <10 h in school children)*.

We found similar results for a detailed examination of the association of adherence and overweight/obesity stratified by the combination of adherence to key messages published in Kovacs et al. (18) (their Table 6, rightmost columns) and the identical analysis restricted to subsamples of T1 or T3 participants (Table 6). However, the majority of CPEs for a restricted subsample of T3 participants were well above 10%.

**Table 6:** Odds ratios with 95% confidence intervals for overweight/obesity stratified by the combination of adherence to key messages regarding media consumption, physical activity, and sleep.

			**Baseline association**										
			**T0 full sample**			**T1 subsample**			**% Change in**	**T3 subsample**			**% Change in**
**TV**	**PA**	**Sleep**	**Normal^a^**	**Obese^b^**	**OR^c^ (95% CI)**	**Normal^a^**	**Obese^b^**	**OR^c^ (95% CI)**	**OR (T1 vs. T0)**	**Normal^a^**	**Obese^b^**	**OR^c^ (95% CI)**	**OR (T3 vs. T0)**
–	–	–	1,289	377	ref.	933	259	ref.	ref.	498	128	ref.	ref.
+	–	–	1,168	317	0.73 (0.61–0.88)	834	215	0.72 (0.58–0.91)	−1.4	444	96	0.66 (0.48–0.92)	−9.6
–	+	–	227	44	0.62 (0.44–0.90)	174	28	0.56 (0.36–0.87)	−9.7	89	16	0.72 (0.40–1.30)	16.3
–	–	+	550	132	0.91 (0.70–1.18)	367	77	0.85 (0.61–1.17)	−6.6	150	35	1.01 (0.62–1.64)	11.0
+	+	–	166	30	0.54 (0.35–0.82)	117	24	0.68 (0.42–1.10)	25.9	54	13	0.92 (0.47–1.78)	70.4
+	–	+	938	180	0.63 (0.50–0.80)	671	120	0.65 (0.49–0.87)	3.2	296	56	0.75 (0.49–1.15)	19.1
–	+	+	123	16	0.48 (0.27–0.83)	81	10	0.49 (0.24–0.98)	2.1	36	6	0.74 (0.29–1.90)	54.2
+	+	+	234	29	0.42 (0.28–0.66)	164	16	0.38 (0.21–0.66)	−9.5	68	6	0.39 (0.16–0.95)	−7.1
*N*			4,695	1,125		3,341	749			1,635	356		

*OR, odds ratio; CI, confidence interval; ref. reference category*.

a *Normal weight including thin*.

b *Obese including overweight*.

c *Odds ratios and confidence intervals for overweight/obesity of single models adjusted for age, sex, and country*.

*+ Adherence (–: non-adherence) to key messages: TV (total screen time), PA (physical activity), and sleep (sleep duration)*.

## Discussion

In line with earlier research our results suggest that higher attrition at the follow-ups was associated with a higher weight status of children, lower children's study compliance, older age, lower parental education, and parent's migration background (2, 3, 21, 22). For a multi-purpose cohort focusing on overweight and obesity, the observed association between weight status and attrition was perhaps to be expected. For instance, children with higher BMI might have felt more uncomfortable having their weight measured (in underwear) at baseline, causing them to refuse participation in follow-ups. Or participation in IDEFICS/I.Family might not have met the expectations of children and/or parents concerning a health study, leading them to leave the cohort that “did not work out for them.” However, while selection effects on children's BMI did occur, they appeared to only slightly distort the distribution at the upper tail, mainly above the 99% percentile.

We found that older children were less likely to take part in the second follow-up as compared to younger ones. In contrast to studies on adults, the consent of both parents and children was required for inclusion into this study, and it has been shown that this makes recruitment particularly challenging (23). In particular it remains unclear to which degree the opinion of parents and/or children were decisive for participating. It is reasonable to assume that, as they get older, children act more autonomously and hence have more say regarding whether or not to participate. As children transit into puberty, they might find epidemiological studies less interesting or might get increasingly uncomfortable with getting examined in underwear. Unfortunately, although puberty status was part of the study protocol at the second follow-up, it was not included at baseline and at first follow-up, rendering it impossible to investigate links between puberty status and attrition.

Furthermore, the association between children's age and attrition might also be influenced by residential mobility, which has been shown to be highly associated with attrition as it can lead to invalid contact data (14, 22, 24). In most of the participating countries, the transition from primary to secondary school happens when children are between 10 and 12 years old (except for Estonia's and Sweden's single structure school systems) and for many of the children, this transition took place between the first and second follow-up. Hence many families might have used this opportunity to relocate, possibly leading to dropouts if the family moved out of the study region or their contact data became invalid.

As participants were free to decide whether or not to take part in individual study modules, we used the study compliance of both parents and children as proxy-measures of motivation. The fact that both parent's and children's study compliance clustered among high values indicates that once people made the decision to take part they completed the study program as a whole. Nevertheless, children's participation was noticeably lower for the collection of the invasive biosamples. Parents were more likely to complete all modules that took place in the study center (general questionnaire, food frequency questionnaire, and medical history), and less likely to complete the take-home questionnaires (24–h dietary recall). This could be due to the fact that the latter questionnaires were more time consuming and involved setting aside additional time for the study.

Unfortunately, it cannot be ruled out that parents of children with certain diagnoses covered by the medical questionnaire might have been more reluctant to complete it to avoid stigmatization. Previous results from the cohort published elsewhere showed that, for instance, the prevalence of ADHD in the cohort was somewhat lower as compared to the whole population (25).

Heterogeneity analyses revealed that countries differed considerably in how well the overall model captured the influence of different predictors on attrition. However, although the heterogeneity between the countries was high in terms of *I*^2^, a closer look using country stratified forest plots revealed that for many predictors all countries showed similar trends. For some predictors, high heterogeneity estimates appeared to be caused by single outliers because excluding these outliers improved *I*^2^ considerably. While there are plausible explanations for some of the deviations from the general trend, for others there are none. For instance, Italy's estimates for the influence of educational level probably deviated because of the small proportion of parents with high educational level in their sample. However, it is not clear why female children in Belgium were more likely to take part in the follow-ups, whereas no such association was obvious for other countries. Similarly, we cannot explain why attrition differed for control and intervention regions in Belgium, but not in other participating countries. Often such inconsistencies can be explained by investigating paradata recorded during recruitment [i.e., information about the process of the data collection (26)] with dedicated documentation systems [e.g., (9, 27)]. Unfortunately paradata were only available for the German study cohort (9), rendering an analysis for the whole cohort impossible. The collection of paradata might thus be especially crucial in multicenter cohort studies, where documentation is often difficult to coordinate between different survey teams operating over long periods of time.

Analysis of selection effects on cross-sectional exposure-outcome associations revealed few effects on point estimates when restricting the full sample at baseline (T0) to participants of the first follow-up (T1). Results on CPEs after restricting the exposure-outcome associations to a subsample of second follow-up participants (T3) were mixed. In particular in the detailed analysis of adherence and overweight/obesity CPEs exceeded 10%, potentially caused by a sharp decline in the number of observations for the subgroups.

## Strengths and limitations

Strengths of our study include the large sample size from an international population and the highly standardized procedures for data collection that were enforced by a central quality control. As noted previously, interpretation of our results would have benefitted if information about puberty status would have been gathered at each time point and more centers would have collected paradata.

## Conclusion

Potential bias in cohort studies induced by attrition may vary according to exposure and outcome (28) and even a high level of attrition may have a limited effect on estimates of associations between exposure and outcome (2, 28, 29). Our results, however suggest that the IDEFICS/I.Family cohort gives valid estimates of the associations of interest.

## Author contributions

HP, FL, TV, MT, DM, GE, SdH, LM, GW, and WA contributed to data collection; ML, HP, WA, and SR designed and implemented the research; ML, HP, WA, and SR analyzed and interpreted the data; ML, and SR drafted the manuscript. All authors discussed the results, critically commented on the manuscript, and gave their final approval to the submitted version of the manuscript.

We are grateful to Florence Samkange-Zeeb for critically reviewing an earlier version of the manuscript.

### Conflict of interest statement

The authors declare that the research was conducted in the absence of any commercial or financial relationships that could be construed as a potential conflict of interest.
